# Clinical and radiological evidence of the recurrence of reversible pegvisomant-related lipohypertrophy at the new site of injection in two women with acromegaly: a case series

**DOI:** 10.1186/1752-1947-6-2

**Published:** 2012-01-10

**Authors:** Vincenzo Rochira, Lucia Zirilli, Chiara Diazzi, Stefania Romano, Cesare Carani

**Affiliations:** 1 Chair of Endocrinology, Department of Medicine, Endocrinology and Metabolism, Geriatrics University of Modena and Reggio Emilia, Azienda USL of Modena, Via Giardini 1355, 41126 Modena, Italy; 2 Unit of Diabetology, Department of Medicine, Endocrinology and Metabolism, Geriatrics University of Modena and Reggio Emilia, Via Giardini 1355, 41126 Modena, Italy

## Abstract

**Introduction:**

Pegvisomant-related lipohypertrophy may revert when changing the site of injection, but the lipohypertrophy may recur at the new site of injection. The strength of evidence, however, is weak and comes from information obtained from physical examination only.

**Case presentation:**

We studied two Caucasian women with acromegaly, aged 51 and 71 years, with pegvisomant-related lipohypertrophy. Our two patients were evaluated at baseline, when the site of pegvisomant injection was the periumbilical abdominal region, and then four months after switching the injection site from the abdomen to both thighs. Both physical examination and radiological studies (magnetic resonance imaging and dual energy X-ray absorptiometry) demonstrated that the abdominal lipohypertrophy progressively reverted in both patients after switching the site of injection to the thighs. However, lipohypertrophy reappeared at the new site of injection. The radiological outcome confirmed the reversibility of pegvisomant-related lipohypertrophy and strengthened the body of evidence on this issue.

**Conclusion:**

In clinical practice, physical examination of the injection site or sites leads to an early detection of lipohypertrophy during pegvisomant treatment. Radiological procedures may be of help to confirm subcutaneous fat changes and for a precise monitoring of fat redistribution. Patients should get appropriate information about lipohypertrophy before starting pegvisomant treatment since the rotation of the site of injection may prevent lipohypertrophy.

## Introduction

The growth hormone (GH) receptor antagonist pegvisomant is effective in the control of acromegaly since it decreases serum insulin-like growth factor 1 (IGF-1) and improves the health status of people with acromegaly [1]. In clinical practice, clinicians recommend pegvisomant for recurrences of acromegaly after transsphenoidal surgery, especially when the primary medical treatment with somatostatin analogues (SSA) is ineffective [1]. Pegvisomant treatment is well tolerated and is considered safe [1] even if we consider long-term studies [1-3]. Clinically relevant side effects include a transient increase of liver enzymes [1,4], an increase in size of the pituitary tumor [1-6] and headache [2]; their frequency is similar to that of other treatment regimens [6]. In 2006, Maffei *et al*. first described a condition of lipohypertrophy at the site of pegvisomant injection in two people with acromegaly [7]. Subsequently, further case series [8,9] and retrospective cross-sectional analyses of larger cohorts of patients treated with pegvisomant [2,3] supplied similar observations. However, the actual pathogenetic mechanism involved in pegvisomant-related lipohypertrophy remains unknown. Both GH-deficiency and the local modulation of lipolytic enzymes, such as 11-β-hydroxysteroid dehydrogenase, may be involved in the development of pegvisomant-related lipohypertrophy [7-9]. In the clinic, pegvisomant withdrawal seems to reverse subcutaneous fat accumulation; however, evidence provided until now has come from observations of a single case report, patients' pictures of the site of fat accumulation [8] and the observation of body changes recorded at physical examination [9]. An evidence-based outcome of lipohypertrophy reversion is lacking since a radiological evaluation before and after switching the site of injection is not yet available in literature [7-9], leaving the strength of the evidence weak at the moment. Notwithstanding the weakness of evidence, however, experts provided some simple clinical recommendations [9].

We document the reversibility of pegvisomant-related lipohypertrophy by a prospective radiological evaluation, thus improving the strength of evidence for this clinical condition.

## Cases presentation

Patient 1 is a 51-year-old Caucasian woman with acromegaly, treated with pegvisomant (Somavert, Pfizer, NY, USA) after three unsuccessful transsphenoidal endoscopic tumor resections and ineffective somatostatin analog treatment. This patient presented to the outpatient clinic of Endocrinology of Modena with the clinical suspicion of acromegaly. A hormonal analysis demonstrated raised serum levels of IGF-1 (666 ng/mL; normal range: 94 ng/mL to 267 ng/mL) and basal GH (22.1 ng/mL) and a pituitary magnetic resonance imaging (MRI) scan disclosed a pituitary macroadenoma with a diameter of 12 mm, which extended to her right cavernous sinus. Our patient underwent three transsphenoidal endoscopic pituitary tumor resections, which did not lead to complete tumor resection because of residual tissue within the right cavernous sinus. She was treated with the somatostatin analog, octreotide long-acting release (LAR) (Sandostatin LAR, Novartis Pharma AG, Basel, Switzerland), at the dosage of 20 mg intramuscularly every 28 days for three months followed by 30 mg every 20 days but this did not lower her IGF-1 serum levels (821.3 ng/dL and 741.3 ng/mL, respectively). Thus, she was started on pegvisomant treatment 13 months after the initial diagnosis of acromegaly, at a dosage of 10 mg/day delivered subcutaneously, which rapidly decreased her IGF-1 serum level (251.3 ng/mL after two months of treatment). In July 2008, our patient underwent pituitary-directed Gamma-Knife stereotaxic radiosurgery. She is still under pegvisomant treatment at a dosage of 10 mg subcutaneously, daily resulting in a good control of her serum IGF-1 (206 ng/mL).

Patient 2 is a 71-year-old Caucasian woman with acromegaly, treated with surgery 23 years before the initiation of pegvisomant treatment; the latter was administered because all previous pharmacological treatments failed. This patient came to our attention with IGF-1 serum levels higher than normal (280.1 ng/mL; normal range: 20 ng/mL to 182 ng/mL) during cabergoline treatment (Dostinex, Pfizer) at a dose of 0.1 mg twice weekly. Our patient reported a previous long history of acromegaly treated with surgery by means of a conventional transnasal transsphenoidal approach. A pituitary MRI showed a small piece of residual adenomatous tissue. Previous pharmacological treatments included bromocriptine, octreotide (Sandostatin, Novartis Pharma AG), octreotide LAR, and lanreotide (Ipstyl, Ipsen, Milan, Italy) but our patient had discontinued somatostatin and SSAs due to intolerance to the drugs; notwithstanding, they were effective in controlling the disease. As the control of the disease was suboptimal (her serum IGF-1 was constantly higher than the upper limit of the normal range) with cabergoline treatment, our patient was switched to pegvisomant therapy at a dose of 10 mg/day subcutaneously. The latter was effective in normalizing her IGF-1 serum levels (150 ng/mL after two months of treatment).

After starting pegvisomant therapy, both women developed a rapid, progressive increase in subcutaneous fat depots at the site of injection in the abdominal periumbilical region. Both patients reported abdominal fat accumulation, and physical examinations performed four and two months after starting pegvisomant treatment for patient 1 and patient 2, respectively, revealed a soft, painless anterior abdominal wall swelling, consisting with a thickening of subcutaneous fat tissue.

Both patients underwent detailed clinical and radiological investigations at baseline (Phase 1), after at least eight months of pegvisomant treatment (10 mg once a day) at the abdominal site, when abdominal lipohypertrophy had just started developing. Soon after the baseline evaluation, both patients switched the injection site from the abdomen to the anterior surface of both thighs. The dose of pegvisomant was not changed. A radiological evaluation was repeated after four months (Phase 2). A hormonal evaluation and study of their body composition by means of a physical evaluation, MRI of the abdomen and thighs and dual energy X-ray absorptiometry (DXA), were performed in Phase 1 and Phase 2 with the aim of detecting and quantifying changes in their subcutaneous adipose tissue.

At physical examination, the abdominal wall swelling decreased progressively in both patients after switching the site of injection to the thighs. Swelling, however, developed at these new sites of pegvisomant injection in both patients within four months.

**Figure 1:**
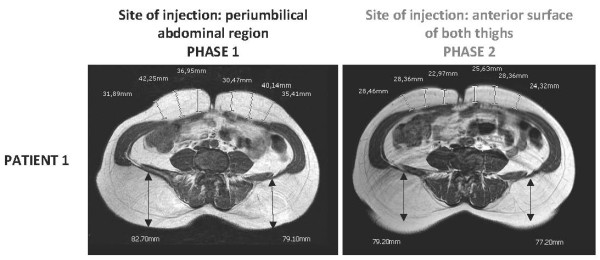
**Abdominal MRI scans of patient 1 when injecting pegvisomant (10 mg/day) subcutaneously in her abdomen (Phase 1) and thighs (Phase 2)**.

**Figure 2:**
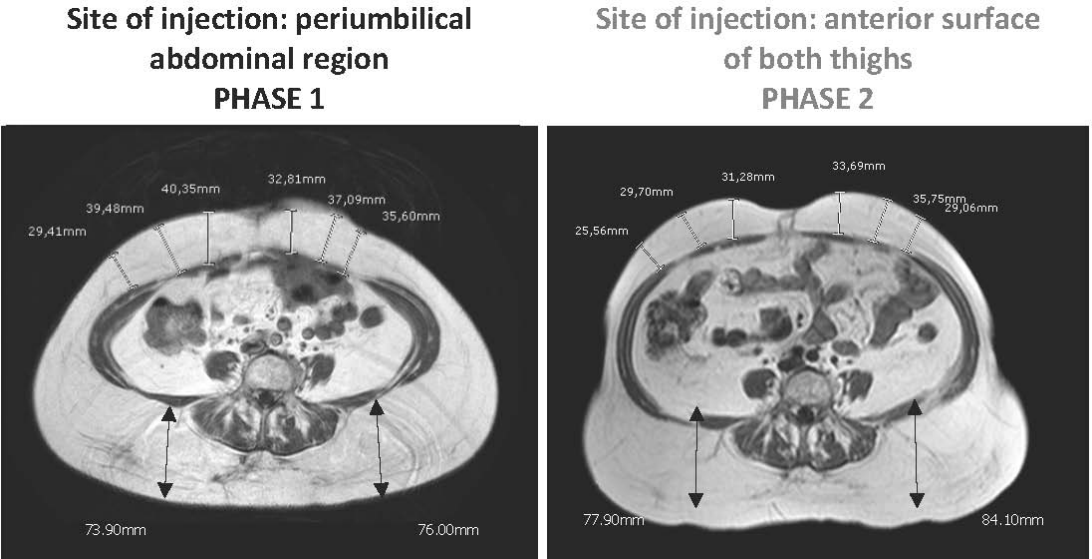
**Abdominal MRI scans of patient 2 when injecting pegvisomant (10 mg/day) subcutaneously in her abdomen (Phase 1) and thighs (Phase 2)**.

**Figure 3:**
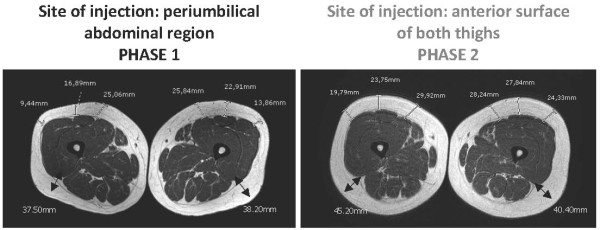
**MRI scans of the thighs of patient 1 when injecting pegvisomant (10 mg/day) subcutaneously in her abdomen (Phase 1) and thighs (Phase 2)**.

**Figure 4:**
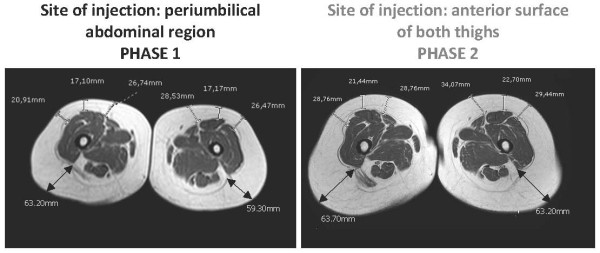
**MRI scans of the thighs of patient 2 when injecting pegvisomant (10 mg/day) subcutaneously in her abdomen (Phase 1) and thighs (Phase 2)**.

The MRI images and evaluation of fat mass content with DXA (Table 1) confirmed a reduction in abdominal subcutaneous fat thickness in patient 1 (Figure 1) and patient 2 (Figure 2), together with a concomitant increase in the subcutaneous fat at the anterior surface of both thighs in patient 1 (Figure 3) and patient 2 (Figure 4). Body fat thickness also increased in the flanks and back areas (only in patient 2; Figure 2) and the posterior surface of the thighs in both patients (Figures 3 and 4).

**Table 1:** Hormonal and adipose tissue measurements by DXA in the two women with acromegaly.

Parameters	Normal range	Patient	Phase 1^a^	Phase 2^b^
**Serum IGF-1 (ng/mL)**	94 to 267 (60.8 to 297.7)^c^	**Patient 1**	264	252
	
	20 to 182 (34.5 to 219)^c^	**Patient 2**	162.0	94

**Serum IGFBP-3 (ng/mL)**	3400 to 6900	**Patient 1**	5670	5550
	
		**Patient 2**	4737	3623

**Body weight (kg)**	-	**Patient 1**	79.0	81.5
	
		**Patient 2**	58.3	59.5

**Abdominal fat on DXA (g)**	-	**Patient 1**	11555	10033
	
		**Patient 2**	14200	13133

**Percentage of abdominal fat on DXA (%)**	-	**Patient 1**	32.3	31.6
	
		**Patient 2**	43.9	43.1

**Thigh fat on DXA (g)**	-	**Patient 1**	8344	9032
	
		**Patient 2**	7883	9692

**Percentage of thigh fat on DXA (%)**	-	**Patient 1**	32	33
	
		**Patient 2**	43.7	47.5

**Total body fat on DEXA (g)**	-	**Patient 1**	25285	27450
	
		**Patient 2**	27321	29212

**Percentage of total body fat on DEXA (%)**	-	**Patient 1**	34.7	36.9
	
		**Patient 2**	44.4	46.8

**Total fat free mass on DEXA (g)**	-	**Patient 1**	47590	47000
	
		**Patient 2**	34190	33149

**Percentage of total fat free mass on DEXA (%)**	-	**Patient 1**	65.3	63.1
	
		**Patient 2**	55.6	53.2

**^a^**Pegvisomant (10 mg/day) administered by subcutaneous injection into the abdomen; ^b^pegvisomant (10 mg/day) administered by subcutaneous injection into the thighs; ^c^age-related ranges from the Laboratory of Modena and the Italian population.

A tendency for a reduction in IGF-1 and IGF-binding protein 3 and a better control of the disease was documented, especially in patient 2 (Table 1). Neither patient experienced other side effects; in particular, no enlargement of the pituitary residual tumor was recorded. Notwithstanding advice by us about the benefits of the treatment and the importance of continuing pegvisomant administration, patient 2 decided to stop pegvisomant because of the discomfort related to lipohypertrophy. In patient 2, pegvisomant withdrawal resulted in a progressive disappearance of the lipohypertrophy. Patient 1 was asked to rotate the site of injection over several districts (thighs, abdomen, arms and buttocks). As a consequence, a minimal swelling developed at these sites, without affecting self-image and patient's compliance to the treatment.

## Discussion

The clinical observation of pegvisomant-related lipohypertrophy is recent and has been documented in a small number of cases [7,8]. In 2008, the group of Melmed based their extensive clinical experience on the description of seven cases. The physical examination of these patients suggested that pegvisomant-related lipohypertrophy may be reversible [9], as previously suggested [8], but that it may develop again at the new site of injection [9]. The latter study is the largest in terms of the number of patients enrolled, but it does not provide objective evidence of pegvisomant-related lipohypertrophy reversibility because of the lack of radiological outcomes. The two cases described here strengthen the evidence on pegvisomant-related lipohypertrophy reversibility as well as on its possible recurrence at the new site of injection. In fact, subcutaneous fat changes documented with MRI provide a highly powerful outcome, thus confirming and reinforcing the knowledge on this clinical condition associated with subcutaneous pegvisomant injection [7-9]. Of note, the occurrence of lipohypertrophy has been observed in 10 women with acromegaly, of the total 12 cases described in detail until now (including the two cases here described) [7-9]. From this cases review, it seems that the risk of developing pegvisomant-related lipohypertrophy is higher in women than in men [9]. Body image changes are of concern, especially in women. This issue is of clinical relevance since, even though lipohypertrophy is not considered a serious adverse event [2-4], it may compromise the patient's compliance to treatment by altering self-image [4,9], as confirmed also by this report. The results from MRI evaluation show that lipohypertrophy of the thighs seems to be quantitatively smaller than that previously developed at the abdomen, probably as a consequence of the shorter period of pegvisomant injection at the thigh sites. Accordingly, the reduction of lipohypertrophy seems to be associated with a better control of IGF-1 serum levels in both patients, especially patient 2 (Table 1). The better control of IGF-1 serum levels occurring during phase 2 in both subjects is further substantiated by the corresponding increase in body weight and total body fat mass as well as by the decrease in total body fat free mass (Table 1). Furthermore, fat redistribution at both the abdominal (Figures 1 and 2) and thighs (Figures 3 and 4), particularly the increase in body fat in the posterior areas of the abdomen and the thighs, during Phase 2 proves a better control of the disease. As suggested by Marazuela *et al*. [8], increased subcutaneous fat at the site of injection may negatively affect pegvisomant absorption. With this in mind, whether or not reducing the frequency of pegvisomant injection [10] or changing the dosage may be successful in preventing lipohypertrophy and ensuring a concomitant good control of the disease requires further elucidation.

## Conclusion

Pegvisomant-related lipohypertrophy develops at the site of injection and it may be reversible, but it could also reappear at the new site of injection. According to the results provided by us and other authors [7-9], practical clinical advice for patients, useful for avoiding the worsening of patient's compliance to treatment, should include appropriate information about the possible occurrence of lipohypertrophy at the injection site and its reversibility, the requirement to rotate the injection site frequently and self-monitoring of changes at the site of injection. Furthermore, the clinical follow-up of the person with acromegaly under pegvisomant treatment should also include physical examination of the injection site or sites for the early detection of lipohypertrophy [9], which might also be performed using radiological procedures (such as ultrasound or MRI), which are useful for monitoring changes in subcutaneous fat.

## Consent

Written informed consent for publication of this case series and any accompanying images was obtained from the patients. Copies of the written consent are available for review by the Editor-in-Chief of this journal.

## Competing interests

The authors declare that they have no competing interests.

## Authors' contributions

VR and SR designed the study. VR, SR and CC performed the clinical examinations. LZ and VR analyzed the data according to the literature and wrote the first draft of the manuscript. CD, SR, VR and LZ contributed to the final version of the manuscript. CC supervised the entire work. All authors read and approved the final manuscript.
